# Examining Nurses' Personality Traits and Work–Life Balance in Türkiye: A Cross-Sectional Study

**DOI:** 10.1155/jonm/2852264

**Published:** 2025-07-28

**Authors:** Büşra Mazak Aşkan, Sevim Şen Olgay

**Affiliations:** ^1^Institute of Health Sciences, Yeditepe University, Istanbul, Turkey; ^2^Department of Nursing, Faculty of Health Sciences, Acıbadem Mehmet Ali Aydınlar University, Istanbul, Turkey

## Abstract

**Background:** Personality is a significant measure that influences an individual's perception of balancing work and family. Understanding an individual's personality can help develop strategies for a healthier work–life balance.

**Aim:** This study aimed to examine the relationship between nurses' personality traits and work–life balance.

**Methods:** This was a descriptive cross-sectional study. The sample comprised 188 nurses working in institutions affiliated with a foundation in Istanbul, Türkiye. Data were collected using a sociodemographic information form, the Five-Factor Personality Inventory (5FPI), and the Work–Life Balance Scale (WLBS). Descriptive statistics, independent group *t*-test, one-way analysis of variance, and regression analysis were used in data analysis. This study was prepared in accordance with the Strengthening the Reporting of Observational Studies in Epidemiology (STROBE) checklist.

**Results:** The work–life balance of nurses was above average (3.23 ± 0.74); among five-factor personality traits, agreeableness and neuroticism had the highest and lowest scores, respectively. Regression analysis showed that personality traits of nurses had a 23.5% effect on work–life balance.

**Conclusions:** The findings showed that organizational factors are significant in nurses' work–life balance levels and that nurses with a dominant neuroticism factor experience work–life balance.

**Implications for Nursing Management:** Nursing management should examine the personality traits of nurses, ensure that their strengths and developmental aspects are discovered, and make plans accordingly. Furthermore, to increase awareness, personal development training, workshops, or courses can be organized at regular intervals.

## 1. Introduction

In the health sector, workload is increasing owing to several factors, including the increasing use of rapidly developing technology, globalization, aging of the population, increasing demand for health services, and lack of qualified health workforce [1–3]. All of these factors lead to work–life conflict as employees spend less time on their families and personal lives [3]. This problem is particularly evident in occupations with intensive working hours or schedules [4]. Work–life balance is the state of balance wherein professional responsibilities and personal life are provided equal weight. This balance is an essential criterion for achieving satisfaction and effective performance in both personal and professional lives while minimizing conflicts between different roles [1, 3, 5–8]. Owing to several challenging working conditions, including high stress and workload, insufficient manpower resources, and long working hours, nurses should increasingly maintain work–life balance [1, 4, 5, 7]. Income status, department, experience, working hours, and shift types are critical factors affecting this balance. However, nurses' working conditions frequently make achieving a healthy work–life balance difficult [8]. Disruption of the balance between work and life can lead to consequences, including decreased job satisfaction, stress, decreased work performance, and absenteeism [4, 9, 10]. Additionally, Ezeobele et al. [11] stated in their study that providing work–life balance for nurses helps reduce stress and lead a healthy life. Studies examined the relationship between nurses' work–life balance and various organizational and psychological factors. These include leadership and job satisfaction [4, 12], employee dissatisfaction [2], presenteeism [13], resilience and burnout [14], job demands and job control [7], turnover intentions and physical distress [15], intention to leave the profession [16], retention intention [17], work–family conflict [5], and perceived workload [18]. These findings highlight the importance of targeted interventions to enhance nurses' work–life balance, ultimately improving both individual well-being and organizational outcomes.

The conservation of resources (COR) theory provides a valuable framework for understanding how individuals manage their work–life balance [19, 20]. According to this theory, individuals are motivated to acquire, preserve, and enhance their resources, with resource loss being a major source of stress [21, 22]. Furthermore, the theory suggests that individuals perceive resource depletion as more psychologically distressing than the positive effect of an equivalent resource gain [19–21, 23]. Moreover, it highlights individuals' efforts to safeguard their resources when facing stress, emphasizing the psychological and emotional consequences of resource depletion [21, 23]. As a result, resource depletion can negatively impact an individual's work–life balance [19–21, 23]. Within this framework, work–life balance depends on an individual's ability to protect and replenish their physical, cognitive, and emotional resources [24]. Additionally, variations in work–life balance among individuals are influenced not only by working conditions but also by personality traits [25]. Personality traits, in turn, shape how individuals manage their resources, influencing their ability to sustain a work–life balance [26].

Personality traits significantly influence how individuals allocate resources between work and personal life, cope with stress, and manage their time [27]. To better understand these influences, the Big Five Personality Traits model provides a widely accepted framework. This model describes personality through five key dimensions: extraversion, agreeableness, conscientiousness, neuroticism, and openness to experience [25, 27, 28]. Each of these traits shapes how individuals perceive situations, regulate their behavior, and establish a balance between work and personal lives [28]. Since priorities, responsibilities, and management skills vary across individuals, work–life balance holds different meanings for everyone [3]. While those with strong organizational and time management skills may navigate this balance more effectively, individuals with a strong work ethic may struggle to set boundaries [28]. Understanding these personality-based differences is essential in developing tailored strategies to promote a healthier work–life balance. This is particularly crucial in professions like nursing, where maintaining work–life balance directly impacts both employee well-being and institutional outcomes [8]. Studies demonstrated that nurses' personality traits influence various factors, including perceived work environment [29], nursing professionalism [30], nursing competence [31], conflict management [32], career alignment [33], life satisfaction [34], stress coping styles [35], burnout [36], job satisfaction [37], compassion fatigue [38], work stress [39], psychological resilience [40], and work–life quality [41]. These findings underscore the importance of examining personality traits in relation to work–life balance, particularly in high-demand professions like nursing, where occupational stress and emotional resilience play a significant role in overall job performance and well-being.

Recognizing the influence of personality on work–life balance management can help healthcare organizations strengthen support systems and develop more effective interventions. Moreover, this research can guide managers in creating tailored strategies to assist nurses in balancing the complex demands of their professional and personal lives, ultimately enhancing job satisfaction, productivity, and overall well-being. However, there is limited research in the literature on the impact of nurses' personality traits on work–life balance. Further studies are needed to gain a more comprehensive understanding of this relationship. This study provides valuable insights for workforce planning and the development of professional support mechanisms. Specifically, it examines the relationship between nurses' personality traits and work–life balance, addressing the following research questions:

1. What are the personality traits of nurses?
2. What is the relationship between nurses' personality traits and their work–life balance?

## 2. Materials and Methods

### 2.1. Study Design

This study employed a cross-sectional and descriptive design and was conducted between April and September 2023 in hospitals affiliated with a foundation in Istanbul, which has a population of approximately 18 million individuals.

### 2.2. Study Participants

The institution where the study was conducted is one of the significant nonprofit foundations providing healthcare services in Türkiye. The study was carried out in two private, tertiary-level hospitals affiliated with this foundation, both located on the European side of Istanbul. The institution has national and international quality certificates. Overall, 500 nurses are employed in this institution. For this universe, which is not a homogeneous structure for the calculation of the required sample size in this study, the required sample size was calculated as *n* = 500 (1.96) 2 (0.2) (0.8)/(0.5) 2 (500 − 1) + (1.96) 2 (0.2) (0.8) = 165 with a 95% confidence interval and ±5% sampling error [42]. The following were the inclusion criteria: (1) working as a nurse, (2) having completed the orientation process, and (3) being a volunteer. Two hundred nurses who met these criteria were invited to the study via e-mail, and 188 nurses filled out all forms completely (94%).

### 2.3. Instruments

Data were collected using the sociodemographic information form, Work–Life Balance Scale (WLBS), and Five-Factor Personality Inventory (5FPI). All instruments were administered in Turkish, the native language of participants. The 5FPI and WLBS had previously been adapted and validated in Turkish, and no further translation was required.

#### 2.3.1. Sociodemographic Information Form

This form was developed by the researcher by scanning the relevant literature. The form comprises 13 questions, including the sociodemographic characteristics of nurses (age, education level, professional experience level, duty, and place of duty), personality traits, and factors affecting work–life balance [43, 44].

#### 2.3.2. WLBS

This scale was developed by Apaydın [45]. A five-point Likert-type rating (5, completely agree; 1, completely disagree) was used in the scale scoring. The scale has the following four subdimensions: work–life harmony (items 6, 7, 8, 9, 17, and 19), neglecting life (items 1, 2, 4, 5, 10, and 11), taking time for oneself (items 12, 13, 18, and 20), and life is all about work (items 3, 14, 15, and 16). It encompasses 20 items, including 7 positive statements and 13 negative statements (1, 2, 4, 5, 10, 11, 12, 13, 14, 15, 16, 18, and 20). An increase in the scale's overall score indicates a positive increase in work–life balance. The overall reliability of the scale was calculated as 0.91, and its subdimensions were 0.88, 0.81, 0.77, and 0.79, respectively [45]. In this study, Cronbach's alpha value was 0.90, and the subdimensions were 0.84, 0.82, 0.89, and 0.88, respectively.

#### 2.3.3. 5FPI

This scale was developed by Somer et al. using Goldberg [46, 47]. This scale comprises 44 items on a five-point Likert-type (1, strongly disagree; 5, strongly agree). This scale has the following five factors: extraversion, neuroticism, agreeableness, conscientiousness, and openness to experience. When establishing factor scores, 16 items (2, 6, 8, 9, 12, 18, 21, 23, 24, 27, 31, 34, 35, 37, 41, and 43) are reverse coded. The factor scores obtained in this manner indicate that the relevant personality trait symptoms are high. As each dimension expresses a different personality trait, the total score of the scale is not taken. Cronbach's alpha value of the scale was calculated between 0.88 and 0.96 [46]. In this study, Cronbach's alpha value ranged between 0.89 and 0.83.

### 2.4. Ethical Considerations

The Yeditepe University Non-Interventional Clinical Research Ethics Committee approved this study (application number, 202304Y0382; date, April 14, 2023). Institutional permissions and scale use permissions were obtained. All participants filled out the informed consent form. The principles of the Declaration of Helsinki were followed in this study.

### 2.5. Study Processes

An online invitation was sent to the e-mail addresses of nurses who met the inclusion criteria in this study. Participants were asked to provide informed consent and confirm that they met the specified eligibility criteria when accessing the online form. The average time to complete the forms was 12–15 min. The Strengthening the Reporting of Observational Studies in Epidemiology (STROBE) checklist was used in this study. To successfully send the survey, all questions should be completed.

### 2.6. Statistical Analysis

Data were evaluated using Statistical Package for the Social Sciences (SPSS) 22. To determine whether the research variables showed normal distribution, kurtosis and skewness values were examined [48, 49]. In the literature, the results of the kurtosis and skewness values of the variables are accepted as normal distribution when they are between +1.5 and −1.5 and between +2.0 and −2.0 [48, 49]. In this context, as the data showed a normal distribution, parametric methods were used. Frequency and percentage analyses were used to determine the descriptive characteristics, and mean and standard deviation statistics were used to examine the scale. Relationships between the dimensions determining the scale levels of the nurses were examined using the Pearson correlation and linear regression analyses [48]. To examine the differences in the scale levels according to the descriptive characteristics of the nurses, independent group *t*-test, one-way analysis of variance, and post hoc (Tukey, LSD) analyses were applied [48]. To calculate the effect size, Cohen (d) and eta-squared (*η*^2^) coefficients were used. The effect size shows whether the difference between the groups is large enough to be considered significant. The Cohen value is evaluated as 0.2 (small), 0.5 (medium), and 0.8 (large); the eta-squared value is evaluated as 0.01 (small), 0.06 (medium), and 0.14 (large) [49].

## 3. Results

The average age of the nurses was 29.10 ± 5.86 years. Of the participants, most were females (87.2%, *n* = 164), single (69.1%, *n* = 130), had a bachelor's degree (72.9%, *n* = 197), and had insufficient income (60.1%, *n* = 113). Most of the nurses working in the institution were assigned in inpatient wards (56.4%, *n* = 106), were staff nurses (61.7%, *n* = 116), had 1–5 years of institutional and professional experience (43.1%, *n* = 81), worked > 10 h (63.3%, *n* = 119), and in mixed day/night shifts (60.6%, *n* = 114) (Table 1).

The WLBS scores of the nurses were above average (3.23 ± 0.74). From the subdimensions of the WLBS, the nurses received the highest mean score in work–life harmony (3.34 ± 0.80) and the lowest mean score in taking time for oneself (2.63 ± 0.92) (Table 2). The mean scores of the personality traits of the nurses were, from high to low, agreeableness (4.01 ± 0.45), conscientiousness (3.85 ± 0.40), openness to experience (3.72 ± 0.46), extraversion (3.65 ± 0.61), and neuroticism (2.54 ± 0.63) (Table 2).

A significant difference was noted between the total WLBS scores of nurses who were married, had children, had a postgraduate education, were responsible/supervisors, had high income, were managers, had > 11 years of experience in the institution and profession, and worked < 10 h at daytime (*p* < 0.05). Additionally, male nurses had higher total WLBS and work–life harmony and lower neglecting life scores (*p* < 0.05). Nurses who were married, high school graduate, working in the emergency department, had > 11 years of experience in the profession, and working < 10 h at daytime had high work–life harmony scores (*p* < 0.05). Low neglecting life scores were observed in those with social and private insurance, married, had children, postgraduate education, being an education/special branch nurse, high income, being a manager, > 11 years of experience in the institution and profession, and continuously worked < 10 h at daytime (*p* < 0.05). Nurses who did not have children, were staff nurses, had low income, worked in intensive care, had 1–5 years of experience in the profession and institution, and worked > 10 h in mixed day/night shifts had high scores for taking time for oneself (*p* < 0.05). Those who were staff nurses, had low income, worked in intensive care, had 1–5 years of experience in the profession and institution, and worked > 10 h in mixed day/night shifts showed high scores for neglecting life (*p* < 0.05, Table 3).

High neuroticism scores were observed among females, single, childless, staff nurses, and nurses working mixed day/night shifts (*p* < 0.05). High extraversion scores were noted among male nurses (*p* < 0.05). High conscientiousness scores were exhibited by responsible/supervisor nurses (*p* < 0.05). High openness to experience scores was observed among nurses with social insurance, single, and childless (*p* < 0.05, Table 4).

Regression analysis explained 23.5% of the variance in the work–life balance level of the personality traits of nurses (*R* = 0.505, *R*^2^ = 0.235, *p* < 0.05). Neuroticism among the personality traits decreased work–life balance and work–life harmony and increased the level of neglecting life, taking time for oneself, and life is all about work. Moreover, the findings revealed that conscientiousness increased the level of neglecting life, extraversion increased the level of taking time for oneself, and openness to experience increased the level of life is all about work. These findings showed that the personality traits of nurses were significant predictors of work–life balance (Table 5).

## 4. Discussion

This study was conducted to investigate the relationship between nurses' personality traits and work–life balance. The health sector follows a rapidly developing and changing world, and this situation has revealed the role of nurses in providing quality patient care and efficient operations [50]. However, difficult working conditions also generate difficulties in maintaining work–life balance. Roth et al. [1] reported that nurses' work–life balance was below average, and Rony et al. [2] found that 56.55% of nurses experienced work–life imbalance. In contrast, the findings of this study showed that nurses' work–life balance was at an average level, which was inconsistent with those reported by Roth et al. [1] and Rony et al. [2], but aligned with those by Özdemir and Söyük [13]. It is important to note that these studies were conducted in different geographical regions and cultural contexts, which may explain the variations in work–life balance findings. This study, conducted with nurses working in a large institution in Türkiye, suggests that cultural and environmental factors may play a role in shaping nurses' experiences of work–life balance.

In this study, nurses scored high on agreeableness and conscientiousness (Table 2). Previous studies on nurses' personality traits have reported similar findings, with agreeableness, conscientiousness, and openness to experience showing the highest mean scores [38, 51]. Conversely, neuroticism had the lowest mean score, aligning with prior research [37, 51–53].

Individuals with positive traits such as agreeableness and conscientiousness generally possess strong interpersonal resources, including trust, cooperation, and reliability [26, 28]. These traits contribute to effective work management and a positive work environment [3]. However, according to COR theory, resource depletion occurs when individuals fail to protect or replenish their resources [21, 22]. While beneficial, excessively high levels of these traits may lead to overextension, making it challenging to maintain a healthy work–life balance [3].

Highly agreeable individuals are trusting, helpful, reliable, and value cooperation and harmonious relationships [51, 54]. However, their tendency to prioritize others' needs may make it difficult for them to refuse extra work, leading to emotional and cognitive exhaustion. In line with COR theory, assertiveness training and boundary-setting strategies can help them conserve resources and maintain balance [3]. Conscientious individuals, known for their determination, organization, and goal orientation, often prioritize work over personal needs [51, 54]. While these traits support productivity, they can also contribute to resource depletion and emotional exhaustion. To counterbalance this, setting clear boundaries and intentionally allocating time for rest and personal activities are essential [3, 55].

Wang et al. [56] emphasized the importance of personality traits, such as conscientiousness and agreeableness, in fostering trust, collaboration, and effective job performance, particularly during public health emergencies. Similarly, individuals with high openness to experience tend to embrace new challenges and contribute to an engaging work environment, supporting both personal and professional growth [3, 56]. However, according to COR theory, these beneficial traits may also lead to overcommitment, making it difficult for individuals to establish firm boundaries between work and personal lives, ultimately resulting in resource depletion if job demands are not carefully managed [19–21, 23]. In the context of nursing, these personality traits can enhance frontline performance during emergencies; however, they may also increase the risk of burnout and work–life imbalance [3, 36, 56]. Therefore, nursing managers should consider these factors when assigning roles, ensuring that job demands align with individual capacities [56]. Leadership support, assertiveness training, and stress management strategies can help nurses maintain a sustainable work–life balance while maximizing their contributions in high-pressure environments [56, 57].

COR theory suggests that individuals strive to acquire, maintain, and protect their resources (e.g., time, energy, social support). When these resources are depleted, individuals experience stress, which negatively affects their work–life balance [19–23]. Regression analysis results indicate that neuroticism significantly predicts work–life balance and all its subdimensions, supporting the idea that neurotic individuals struggle with resource conservation. Consistent with COR theory, individuals high in neuroticism often experience greater emotional exhaustion and resource loss, making it more difficult for them to effectively manage stress and replenish their psychological reserves [3, 36, 38]. Neurotic individuals tend to be anxious, pessimistic, prone to depression, angry, and restless—traits that contribute to poor coping skills and rapid depletion of emotional and cognitive resources [31, 41, 58]. This difficulty in resource management hinders their ability to detach from work-related concerns, further impairing their work–life balance [3]. Research has shown that nurses with low neuroticism and high extraversion exhibit greater psychological resilience, more effective stress management, and lower susceptibility to burnout, as they are better able to maintain and restore their resources [36, 38, 56]. Sharma's study underscores that work–life balance is a dynamic process requiring self-awareness and adaptability [3]. By leveraging their dominant personality traits while mitigating their weaknesses, individuals can optimize resource allocation and enhance balance between work and personal lives. Wang et al. [56] further emphasize that prioritizing nurses with lower neuroticism and higher conscientiousness and agreeableness in demanding work environments may enhance resource conservation, strengthen teamwork dynamics, and contribute to overall workplace sustainability. Therefore, setting clear boundaries between work and personal lives, prioritizing tasks, managing time efficiently, developing stress-coping strategies, and seeking social support are crucial for preserving and replenishing resources. Implementing these strategies can help mitigate the adverse effects of resource depletion and promote a more sustainable work–life balance, particularly in high-stress professions such as nursing.

## 5. Limitations

The findings of this study were limited to the opinions of nurses working in institutions belonging to a foundation, who constituted the sample and volunteered to participate in this study. Therefore, the results may not be fully generalizable to nurses working in different healthcare settings, both nationally and internationally. Additionally, as this study specifically focuses on the nursing profession, its applicability to other healthcare professionals or occupational groups remains uncertain. From a methodological perspective, the study has limitations related to sampling, as participation was voluntary and restricted to a specific institutional setting. Furthermore, potential biases associated with self-reported data and the impact of pandemic-related workload on survey completion should be taken into account when interpreting the findings.

## 6. Conclusions

This study analyzed the personality traits affecting work–life balance. The personality trait of neuroticism more significantly affects work–life balance than all other personality traits. This study also observed that the work–life balance of nurses is influenced by gender, education, experience, income status, marital status, service they work in, working hours, and shift type. Moreover, it revealed that personality traits are significant factors affecting work–life balance. Nursing work environments encompass critical elements, including professional identity, organizational structure, position within the team, group relations, and leadership dynamics [29, 59]. These components are closely related to the personality traits of nurses and have a critical impact on their practice and job performance [56]. A high level of fit exists between the personality traits of nurses and the work environment and job requirements [56]. This fit may ultimately contribute to the promotion of positive outcomes among nurses, such as work–life balance, job satisfaction, resilience, and psychological well-being. Therefore, leaders should examine the personality traits of nurses and ensure that their strengths and developmental aspects are discovered. Furthermore, organizing activities such as personal development training, workshops, and courses to ensure awareness of developmental aspects and considering these characteristics in assignments can help ensure work–life balance. Lastly, it is recommended that quantitative studies with large sample sizes focusing on human resources regarding the personality traits of nurses and qualitative studies be conducted to thoroughly investigate the subject.

The findings of this study have significant managerial, theoretical, academic, and societal implications.

### 6.1. Managerial Implications

Healthcare administrators and policymakers should consider nurses' personality traits when developing work–life balance strategies. For instance, extraverted nurses may thrive in collaborative team environments, whereas introverted nurses might require quiet spaces and flexible scheduling to maintain well-being. Additionally, nurses with lower emotional stability could benefit from targeted stress management programs, while highly conscientious nurses may require workload regulation to prevent excessive burden. Implementing personality-based interventions can enhance job satisfaction, reduce burnout, and improve retention rates—key factors in addressing ongoing workforce challenges in healthcare.

### 6.2. Theoretical Contributions

This study advances the literature by providing a nuanced understanding of how personality traits shape nurses' ability to balance professional and personal demands. Future research should explore the contexts in which specific personality traits exert stronger effects, such as intensive care units, surgical wards, or community health nursing. Additionally, investigating moderating factors—such as organizational support and leadership styles—may further clarify the relationship between personality traits and work–life balance, offering deeper theoretical insights.

### 6.3. Academic Contributions

Postpandemic research on nurses' work–life balance remains limited, particularly regarding the role of individual personality traits. This study addresses this gap by demonstrating that certain personality profiles are more susceptible to work–life conflicts. The findings underscore the importance of integrating personality-based stress management and self-care strategies into nursing education curricula to better prepare future nurses for the demands of professional practice.

### 6.4. Societal Implications

Enhancing nurses' work–life balance directly contributes to improved patient care quality and safety. For instance, nurses with lower emotional stability may struggle with job-related stress, making psychological support programs essential for maintaining optimal patient care. Similarly, highly conscientious and responsible nurses may be at a greater risk of excessive workload and burnout, necessitating tailored workload management strategies. Recognizing and addressing these personality-driven challenges can foster better healthcare outcomes, greater job satisfaction, and long-term workforce sustainability.

## Figures and Tables

**Table 1:** Demographic and clinical characteristics of the nurses participating in this study.

Variables			
Age	Mean = 29.10 ± 5.86; Min = 21; Max = 49		

		**N** ^∗^	**%**

Gender	Female	164	87.2
	Male	24	12.8

Insurance	Social insurance	38	20.2
	Private insurance	150	79.8

Marital status	Married	58	30.9
	Single	130	69.1

Having a child	Yes	42	22.3
	No	146	77.7

Education status	High school	21	11.2
	Bachelor degree	137	72.9
	Postgraduate degree	30	16.0

Position	Nurse	116	61.7
	Education/special branch nurse	15	8.0
	Team leader nurse	36	19.1
	Responsible/supervisor nurse	21	11.2

Income status	Income is less than expenses	113	60.1
	Income equals expenses	60	31.9
	Income is more than expenses	15	8.0

Department of work	Inpatient service	106	56.4
	Emergency department	14	7.4
	Policlinic	38	20.2
	Management unit	15	8.0
	Intensive care unit	15	8.0

Working time in the profession	< 1 year	22	11.7
	1–5 years	81	43.1
	6–10 years	47	25.0
	≥ 11 years	38	20.2

Working time in the institution	< 1 year	35	18.6
	1–5 years	87	46.3
	6–10 years	43	22.9
	≥ 11 years	23	12.2

Daily working hours	8–10 h	69	36.7
	≥ 10 h	119	63.3

Shift type	Day and night (mixed)	114	60.6
	Day	74	39.4

^∗^Frequency.

**Table 2:** 5FPI and WLBS scores of nurses.

Scales and their subdimensions	Mean ± SD	Min	Max	Cronbach's alpha
WLBS				
Total scores	3.23 ± 0.74	1.50	4.75	0.90
Work–life harmony	3.34 ± 0.80	1.33	5.00	0.84
Neglecting life	2.91 ± 0.92	1.00	5.00	0.82
Taking time for oneself	2.63 ± 0.92	1.00	4.75	0.89
Life is all about work	3.12 ± 0.83	1.50	5.00	0.88
5FPI				
Neuroticism	2.54 ± 0.63	1.12	14.38	0.84
Extraversion	3.65 ± 0.61	1.38	4.88	0.83
Agreeableness	4.01 ± 0.45	2.56	4.89	0.86
Conscientiousness	3.85 ± 0.40	2.67	4.56	0.85
Openness to experience	3.72 ± 0.46	2.20	4.80	0.89

Abbreviations: 5FPI = Five-Factor Personality Inventory, SD = standard deviation, WLBS = Work–Life Balance Scale.

**Table 3:** WLBS and subdimension scores of nurses according to demographic variables.

	Work–life harmony		Neglecting life		Taking time for oneself		Life is all about work		Total score	
	t/F	*p*	t/F	*p*	t/F	*p*	t/F	*p*	t/F	*p*
Gender	3.555	0.000^∗^	−2.016	0.045^∗∗^	−0.112	0.911	−0.409	0.683	2.221	0.028^∗∗^
d	0.777		0.440		—		—		0.485	
*η* ^2^	0.064		0.021		—		—		0.026	

Insurance	1.794	0.074	0.972	0.333	1.460	0.146	2.231	0.027^∗∗^	−0.458	0.648
*η* ^2^	—		—		—		0.022		—	

Marital status	2.048	0.042^∗∗^	−0.658	0.512	−1.336	0.183	−3.959	0.000^∗^	2.223	0.027^∗∗^
d	0.323		—		—		0.625		0.351	
*η* ^2^	0.022		—		—		0.078		0.026	

Having a child	1.904	0.058	−1.183	0.238	−2.930	0.004^∗^	−3.652	0.000^∗^	2.902	0.002^∗^
d	—		—		0.513		0.639		0.508	
*η* ^2^	—		—		0.044		0.067		0.043	

Education	5.275	0.006^∗^	3.020	0.051	1.776	0.172	8.834	0.000^∗^	5.877	0.003^∗^
*η* ^2^	0.054		—		—		0.087		0.060	

Position	1.546	0.204	3.175	0.025^∗∗^	6.116	0.001^∗^	9.315	0.000^∗^	6.131	0.001^∗^
*η* ^2^	—		0.049		0.091		0.132		0.091	

Income	2.238	0.110	5.809	0.004^∗^	5.654	0.004^∗^	8.955	0.000^∗^	8.726	0.000^∗^
*η* ^2^	—		0.059		0.058		0.088		0.086	

Department	3.107	0.017^∗∗^	3.692	0.006^∗^	4.527	0.002^∗^	9.129	0.000^∗^	5.903	0.000^∗^
*η* ^2^	0.064		0.075		0.090		0.166		0.114	

Professional experience	3.018	0.031^∗∗^	2.758	0.044^∗∗^	5.987	0.001^∗^	7.917	0.000^∗^	6.451	0.000^∗^
*η* ^2^	0.047		0.043		0.089		0.114		0.095	

Institution experience	1.519	0.211	3.182	0.025^∗∗^	7.512	0.000^∗^	8.510	0.000^∗^	6.270	0.000^∗^
*η* ^2^	—		0.049		0.109		0.122		0.093	

Working hours	2.034	0.043^∗∗^	−3.354	0.001^∗^	−4.054	0.000^∗^	−8.110	0.000^∗^	5.290	0.000^∗^
d	0.308		0.507		0.613		1.227		0.801	
*η* ^2^	0.022		0.057		0.081		0.261		0.131	

Shift	−3.097	0.002^∗^	3.975	0.000^∗^	5.363	0.000^∗^	7.933	0.000^∗^	−6.287	0.000^∗^
d	0.462		0.593		0.801		1.184		0.938	
*η* ^2^	0.049		0.078		0.134		0.253		0.175	

*Note:* t = independent group *t*-test; F = ANOVA test; d = Cohen; *η*^2^ = eta-squared coefficients.

^∗^ *p* ≤ 0.001.

^∗∗^ *p* < 0.05.

**Table 4:** 5FPI scores of nurses according to demographic variables.

	Neuroticism		Extraversion		Agreeableness		Conscientiousness		Openness to experience	
	t/F	*p*	t/F	*p*	t/F	*p*	t/F	*p*	t/F	*p*
Gender	−2.741	0.007^∗^	3.030	0.003^∗^	−0.077	0.951	0.340	0.734	0.713	0.477
d	0.599		0.662		—		—		—	
*η* ^2^	0.039		0.047		—		—		—	

Insurance	−0.289	0.773	0.783	0.435	1.671	0.096	0.606	0.545	2.494	0.014^∗∗^
*η* ^2^	—		—		—		—		0.057	

Marital status	−2.206	0.029^∗∗^	−1.574	0.117	−1.716	0.088	−0.935	0.351	−3.784	0.000^∗^
d	0.348		—		—		—		0.597	
*η* ^2^	0.025		—		—		—		0.071	

Having a child	−2.205	0.029^∗∗^	−0.172	0.863	−1.328	0.262	0.308	0.758	−2.822	0.005^∗^
d	0.386		—		—		—		0.494	
*η* ^2^	0.025		—		—		—		0.041	

Education	0.581	0.560	2.740	0.067	0.264	0.768	0.940	0.392	0.551	0.577

Position	2.666	0.049^∗∗^	2.068	0.106	2.458	0.064	3.856	0.010^∗∗^	0.520	0.669
*η* ^2^	0.042		—		—		0.059		—	

Income	2.496	0.085	0.530	0.590	1.550	0.215	1.773	0.173	1.513	0.223

Department	1.036	0.390	2.528	0.042^∗∗^	1.211	0.308	1.257	0.289	1.404	0.235
*η* ^2^	—		0.052		—		—		—	

Professional experience	2.249	0.084	1.477	0.222	2.068	0.106	1.698	0.169	0.255	0.857

Institution experience	1.215	0.306	0.656	0.580	1.842	0.141	1.463	0.226	1.337	0.264

Working hours	−1.387	0.167	0.985	0.326	−0.512	0.609	0.459	0.647	−1.207	0.264

Shift	3.478	0.001^∗^	−1.367	0.173	−0.595	0.552	−1.721	0.087	1.393	0.165
d	0.519		—		—		—		—	
*η* ^2^	0.061		—		—		—		—	

*Note:* t = independent group *t*-test; F = ANOVA test; d = Cohen; *η*^2^ = eta-squared coefficients.

^∗^ *p* ≤ 0.001.

^∗∗^ *p* < 0.05.

**Table 5:** Linear regression analysis of personality traits affecting nurses' WLBS total score and subdimensions.

Variables	B	SE	β	t	*p*	95.0% CI	
*Total*⁣*score*^*a*^						*Lower*	*Upper*
Fixed	5.283	0.711		7.428	0.000	3.880	6.686
Neuroticism	−0.627	0.088	−0.539	−7.090	0.000^∗^	−0.801	−0.452
Extraversion	0.010	0.104	0.009	0.099	0.921	−0.195	0.216
Agreeableness	0.016	0.136	0.010	0.115	0.909	−0.253	0.284
Conscientiousness	−0.206	0.168	−0.112	−1.224	0.222	−0.538	0.126
Openness to experience	0.066	0.122	0.041	0.540	0.590	−0.175	0.306

*Work* − *life*⁣*harmony*^*b*^							
Fixed	3.877	0.755		5.137	0.000	2.388	5.366
Neuroticism	−0.574	0.094	−0.454	−6.123	0.000^∗^	−0.759	−0.389
Extraversion	0.169	0.110	0.128	1.533	0.127	−0.049	0.387
Agreeableness	−0.008	0.144	−0.005	−0.056	0.955	−0.293	0.277
Conscientiousness	0.015	0.179	0.008	0.087	0.931	−0.337	0.368
Openness to experience	0.076	0.129	0.044	0.591	0.556	−0.179	0.331

*Neglecting*⁣*life*^*c*^							
Fixed	0.239	0.922		0.259	0.796	−1.580	2.058
Neuroticism	0.691	0.115	0.478	6.027	0.000^∗^	0.465	0.917
Extraversion	−0.015	0.135	−0.010	−0.111	0.912	−0.281	0.251
Agreeableness	−0.024	0.177	−0.012	−0.138	0.890	−0.373	0.324
Conscientiousness	0.449	0.218	0.196	2.060	0.041^∗∗^	0.019	0.880
Openness to experience	−0.178	0.158	−0.089	−1.126	0.262	−0.489	0.134

*Taking*⁣*time*⁣*for*⁣*oneself*^*d*^							
Fixed	0.878	0.906		0.969	0.334	−0.910	2.666
Neuroticism	0.693	0.113	0.479	6.155	0.000^∗^	0.471	0.915
Extraversion	0.297	0.133	0.197	2.244	0.026^∗∗^	0.036	0.559
Agreeableness	−0.229	0.174	−0.113	−1.319	0.189	−0.571	0.113
Conscientiousness	−0.039	0.214	−0.017	−0.180	0.857	−0.462	0.384
Openness to experience	−0.007	0.155	−0.004	−0.048	0.962	−0.314	0.299

*Life*⁣*is*⁣*all*⁣*about*⁣*work*^*e*^							
Fixed	−1.259	0.859		−1.467	0.144	−2.953	0.435
Neuroticism	0.385	0.107	0.294	3.610	0.000^∗^	0.175	0.596
Extraversion	−0.014	0.126	−0.010	−0.108	0.914	−0.261	0.234
Agreeableness	0.300	0.164	0.163	1.822	0.070	−0.025	0.624
Conscientiousness	0.254	0.203	0.123	1.251	0.213	−0.147	0.655
Openness to experience	0.342	0.147	0.190	2.324	0.021^∗∗^	0.052	0.632

*Note: p* = statistical significance; β = regression coefficient; t = the size of the difference relative to the variation in sample data.

Abbreviation: CI = confidence interval.

^a^ *R* = 0.505, *R*^2^ = 0.235, *F* = 12.476, *p* ≤ 0.001, Durbin Watson = 0.551.

^b^ *R* = 0.539, *R*^2^ = 0.271, *F* = 14.925. *p* ≤ 0.001, Durbin Watson = 1.026.

^c^ *R* = 0.434, *R*^2^ = 0.166, *F* = 8.462, *p* ≤ 0.001, Durbin Watson = 0.337.

^d^ *R* = 0.466, *R*^2^ = 0.196, *F* = 10.108, *p* ≤ 0.001, Durbin Watson = 0.977.

^e^ *R* = 0.376, *R*^2^ = 0.118, *F* = 5.988, *p* ≤ 0.001, Durbin Watson = 1.240.

^∗^ *p* ≤ 0.001.

^∗∗^ *p* < 0.05.

## Data Availability

The data that support the findings of this study are available from the corresponding author upon reasonable request.
